# Unusual Presentation of COVID-19 Headache and Its Possible Pathomechanism

**DOI:** 10.7759/cureus.29358

**Published:** 2022-09-20

**Authors:** Bob Daripa, Scott Lucchese

**Affiliations:** 1 Internal Medicine/Neurology, Singapore General Hospital, Singapore, SGP; 2 Medicine, Grant Medical College and Sir J.J. (Jamsetjee Jeejeebhoy) Group of Government Hospitals, Mumbai, IND; 3 Neurology/Headache, University of Arkansas for Medical Sciences (UAMS), Little Rock, USA; 4 Neurology/Headache, University of Missouri School of Medicine, Columbia, USA

**Keywords:** trigeminal ganglion, trigeminal nucleus caudalis, hypothalamus, pathomechanism, ace2, cgrp, headache, covid-19

## Abstract

Headache was the most common neurological symptom during the H1N1 pandemic in 2009 and the most recrudescing symptom of human coronavirus (hCoV) in 2016. Even in this prevailing global coronavirus disease 2019 (COVID-19) pandemic, the main neurological symptom is found to be a headache. Headache phenotypes identified with COVID-19 are largely migraine, tension-type headache, or cough headache located in the frontotemporal or occipital region with wavering intensity and essentially of acute onset. We present two cases of unusual headache phenotypes with COVID-19 infection and attempt to shed light on their pathomechanism. Trigeminal autonomic cephalgia may be a possibility in our case, triggered by the virus itself, either directly or through an indirect path elaborated well in the pathomechanism segment. Severe acute respiratory syndrome coronavirus 2 (SARs-CoV-2) binds to angiotensin-converting enzyme 2 (ACE2) located in the peripheral nerve and intracranial vascular endothelium, sensitizing the trigeminovascular system by further interacting with higher cortical pain centers via the thalamic and hypothalamic nuclei, producing pain. CSF analysis along with opening pressure measurement in Case 2 may portray a comprehensive understanding of our patient’s headache. Coupling with the dorsal pons and trigeminal nucleus caudalis (TNC), the hypothalamus could be the supreme generator for an attack. Hypothalamic perturbance could be a possible phenomenon for abnormal headache experiences and requires further validation. The possible COVID-19 pain pathway pathomechanism engaging interleukin (IL)-1, IL-6, and tumor necrosis factor (TNF) alpha aided with a cortical spreading depression disturbing the hypothalamus is also described in this study. Undoubtedly, this pandemic could prove to be a guiding tool for mankind, for a comprehensive understanding of the enigmatic concepts of headaches.

## Introduction

The main neurological symptom of the prevailing coronavirus disease 2019 (COVID-19) global pandemic is headache [1]. The pandemic originated in Wuhan, China, in December 2019 due to the severe acute respiratory syndrome coronavirus-2 (SARs-CoV-2) [1]. Astoundingly, the headache was the kingpin of neurological symptoms even in 2009 during the H1N1 pandemic [2] and the most recrudescing symptom of human coronavirus (hCoV) in 2016 [2]. The third edition of the International Classification of Headache Disorders (ICHD-3) ascribes headache to the systemic viral infection (code 9.2.2) without meningitis or encephalitis [1] but, regrettably, has no well-defined constitution of evolving signs and symptoms as portrayed by this positive single-stranded crown-like RNA virus [3].

This pandemic has affected millions and resulted in hundreds of thousands of deaths worldwide [4]. It is also particularly concerning for headaches as evidenced by a study, where 179 hospitalized COVID-19 pneumonic patients with headaches recorded mortality of 23.8% compared to only 7.6% in the non-headache group; however, it seems pertinent to systemic complications [4]. Although COVID-19-related headache prevalence studies across the globe have variable results, ranging from 8%-70.3% [3-6], it has the same, common impact in terms of patients’ suffering. We present two cases of unusual headache phenotypes with COVID-19 infection and attempt to shed some light on its pathomechanism. Well-informed and detailed written consent was acquired from the patients for the publication of these reports.

## Case presentation

Case 1

A 28-year-old female presented with altered olfactory and taste symptoms. The oropharyngeal COVID-19 reverse transcription-polymerase chain reaction (RT-PCR) test was positive and blood parameters were within the normal range. She had no other co-morbid disease and was not on any drugs/pills. On the first day in the clinic, she complained of anosmia and ageusia associated with mild fever. She suffered from a runny nose, back pain, and myalgia, suggestive of viral symptoms. The fever continued for the next seven days and responded well with oral paracetamol 650 mg SOS.

On Day 3, she experienced tiresome intranasal burning pain, which was intermitted and lasted for a very short period (30 seconds to 1 minute). The frequency of such pain was around five to six times per day. There were no provoking factors, and it stayed for the next two to three days before spontaneous recovery. There were neither ophthalmologic signs and symptoms nor any features suggestive of meningitis. On Day 4, multiple episodes of short-lasting (1-2 minutes) intense facial pain appeared, which persisted for another three to four days. She described this pain as if someone was pulling her face from within, admixed with a sense of squeezing. During acute episodes of such facial pain, the patient quit her activities and preferred to lie down. No associated phonophotophobia was observed, and nor were any aggravating or relieving factors reported. The patient noted an intermittent nonproductive and mild dry cough during these days but no sinus tenderness. The lungs were clear on auscultation. The patient was on oral doxycycline along with antihistamine, vitamin C, and zinc supplements with oral mucolytic.

On Day 7, there was an abrupt onset of heaviness in the head, predominantly bilateral and high parietal, which was not related to posture or fever. This stayed for a few hours and appeared once every couple of days and continued for the following three weeks. No blurry vision, neck stiffness, or nausea was reported. She observed remission of her facial sensory symptoms gradually over a few days during this time. Vitals were stable with normal oxygen saturation. CT imaging of the brain was normal with no meningeal enhancement. The patient did not consent to an invasive procedure such as a lumbar puncture. This parietal headache lasted for the next three weeks before spontaneous resolution. A follow-up was conducted after a month, which demonstrated the complete resolution of her symptoms with no residual deficit.

Case 2

A 45-year-old male presented with upper respiratory symptoms of rhinitis and mild productive cough. No shortness of breath or any co-morbid disease was reported. The nasopharyngeal COVID-19 RT-PCR test was positive. In the first two days, the patient had constitutional symptoms of fever, runny nose, and body ache, which responded well to paracetamol 650 mg SOS. On Day 3, the emerged bifrontal head heaviness continued for a few hours during the day. No related photophobia, phonophobia, nausea, vomiting, or neck stiffness was observed. Mild fever with rhinitis was present, but no sensory symptom of anosmia or ageusia was seen. The patient’s headache partially responded to oral paracetamol 650 mg and oral naproxen 500 mg SOS. Diagnostic investigation revealed thrombocytopenia (platelet count 90,000/ml) and mild hyponatremia (sodium 132 mmol/L). The remaining blood parameters, along with C reactive protein, ferritin, and D-Dimer, were within the normal range.

Gradually, the mild fever settled in two weeks’ time; however, the bifrontal headache admixed with a heaviness persona persisted, unrelated to body posture or diurnal variation. On the tenth day, the bifrontal pain augmented to holocranial heaviness and was related to postural variation. It escalated while bending forward, but there was no related giddiness, fainting, ear fullness, or blurry vision. There was no nasal stuffiness, and the headache persisted throughout the day, moderately interfering with the patient’s routine work. No other neurological signs or symptoms were observed. On Day 14, the patient perceived a new quality of headache coexisting with the above-described headache. This novel headache was experienced almost daily, with a tight band-like sensation, mainly felt in the bilateral temporo-occipital region and stayed for one to two hours in the evening. There was no neurological deficit on examination, and ophthalmologic evaluation did not reveal papilledema. Oxygen saturation, along with other parameters, was found to be normal. Contrast CT scan brain ruled out meningeal enhancement or bleeding. The patient did not consent to any invasive procedure, such as lumbar puncture, as he believed that he was not critically ill.

After two months, a follow-up visit was conducted. The headache was persistent, although not continuous, and did not disturb his routine activities. He tried paracetamol, naproxen, topiramate, and a combination of paracetamol with caffeine, ibuprofen, tramadol, and ketorolac, with almost no complete symptomatic benefit. He observed that the intensity of headaches gradually diminished over time. The patient claimed that he became symptom-free in the following two months with no residual deficit.

## Discussion

Headache phenotypes identified with COVID-19 include migraine, tension-type headache, or cough headache [5] located in the frontotemporal or occipital region with wavering intensity and essentially acute onset [1,6]. Trigeminal autonomic cephalgia may be a possibility in Case 1, triggered by the virus itself, either directly or through an indirect path elaborated below in the pathomechanism segment. The trigeminal ganglion receives its sensory C and A-delta fibers projected from the head and face while its other arm receives input from intracranial deeper structures viz. dura and blood vessels located outside the blood-brain barrier. This results in peripheral sensitization, which further triggers central sensitization and facilitates the pain pathway. Cerebrospinal fluid (CSF) analysis, as well as opening pressure measurement in Case 2, could have portrayed some comprehensive understanding of the patient’s headache. The possibility of CSF pressure-related headaches cannot be ruled out. Many studies have not conducted CSF studies, ophthalmoscopic examination, and neuroimaging to rule out meningitis, encephalitis, or cerebrovascular disorders, owing to the precautionary measures of COVID-19 pandemic or the non-presence of any warning sign symptoms related to headache.

For a better understanding of headaches associated with COVID-19, a title search on electronic database engines, namely, PubMed and Google Scholar from December 2019 to December 2021 was conducted using keywords such as ‘Headache and COVID-19’, ‘headache associated with COVID-19’, and ‘headache phenotypes with COVID-19’. Inclusion criteria were a detailed description and classification of headache semiology according to the International Classification of Headache Disorder 3 (ICHD-3). This search also incorporated a pertinent supporting laboratory investigation. Exclusion criteria were roughly similar in most studies viz. impaired consciousness, acute confusional state, meningeal/focal neurological signs, previous cognitive impairment, or traumatic brain injury. The extracted data from various studies are well illustrated in Table 1.

**Table 1:** The tabular format displays different phenotypes of COVID-19-related headaches and key findings if present n: total number of subjects included; n: subjects experiencing headache; a/w: associated with; yrs: years; CSF: cerebrospinal fluid; MRV: magnetic resonance venography; MRA: magnetic resonance angiography; GON: greater occipital nerve; TTH: tension-type headache; CGRP: calcitonin G-related neuro peptide; CRP: C reactive protein; IL-6: interleukin-6

S. no.	Study detail	n	Location of headache	Character of headache and %	Associated symptoms/ triggers	Progression of symptom	Investigations, laboratory trends, and related findings	Key elements/Outcomes Inference and drawbacks	Reference
1	Edoardo Caronna et al.,Spain. Sept 2020. Prospective study. N = 130	n = 74.6% (97/130), Female = 57.7%, Mean age = 50.6±15.3	b/l frontal 47.4%, Holocranial 38.1%, Fronto-cervical 6.2%, Hemicranial 5.2%, Cervical 4.1%	Pressing 70.1%, Throbbing 19.6%, Drilling 5.2%, Shooting 4.1%, Burning 1.0%.	Nausea, vomiting, photophobia, phonophobia, daily pain, worsening with movements, anosmia, ageusia, dizziness, vertigo, neck stiffness	After six weeks: 37.8% (28 out of 74 follow-up patients) had an ongoing, persistent headache	Stable or low level of IL-6 and LDH was noted with headache	Stable or low level of IL-6 and LDH was noted with the headache, but D-dimer, ferritin, and CRP depict the same trend with or without headache. Headache not related to fever. Responded to acute treatment. Efficacy: (Yes = 58.6%, No = 41.4%)	[3]
						17 out of 28, 60.7% had a daily constant headache	D-dimer, ferritin, and CRP show the same trend with or without a headache	The duration of COVID-19 disease in headache subjects was 23.9±11.6 days (compared to 31.2±12 days in non-headache COVID-19 patients)	
								Noted no difference in mortality in the headache group. They interpreted that different mechanisms might work, explaining the different qualities of headaches in different circumstances.	
2	Pedro Augusto et al. Brazil. Sept 2020. Cross-sectional study. N = 73	n = 64.4% (47/73). Median age = 56 years Male= 59.6% (28/47)	Frontal 80%, Temporal 55%, Parietal 49%, Occipital 36%, Bilateral 94%.	B/l migraine phenotype (51%), TTH like 40%	Anosmia or hyposmia = 38.4%	No	No	No scans were done, and no cerebrospinal fluid (CSF) analysis was done. 80% of patients self-evaluated that the current headache is different from previous headaches.	[5]
		Prior headache history; Migraine or TTH: total 64%		Cough-related headache (16.4% total): Cough headache = 4, cough triggering migraine = 4, cough trigger TTH = 4.	Hypogeusia or ageusia = 39.7%			Anosmia/ hyposmia lasted for a median of 10 days (6-17.5 days). Hypogeusia or ageusia (39.7%) lasted for a mean of 5 days (4.5–8.5 days). No difference in gender. Some subjects’ anosmia persisted for >3 months.	
								New daily persistent headaches were noted in 11 subjects.	
3	Rehab et al. Egypt. 2020. Cross-sectional study. N = 172	Headache, n = 172/172	Diffuse = 52.9%, temporal = 18%, Frontal = 23.3%, occipital = 5.8%	Pressing = 40.7%, exploding = 26.2%, Dull = 16.9%, Throbbing = 16.3%	When a/w fever, headache intensity, and frequency were higher	No	CRP done. Higher CRP usually decreases pain threshold 6(37) but in this study, CRP had no association with headache frequency or intensity	Scans: not done	[1]
		Median age = 33		Intensity: Mild = 67.4%, moderate = 24.4%, severe = 8.1%	When a/w dehydration, only headache frequency was higher			A high neutrophil/lymphocyte ratio is seen in migraine attacks 6(40) but no relation found with lymphocytes level	
		Male = 37.2%, Female = 62.8%						No association with ferritin levels or D-dimer levels (although D-dimer is found to be high in migraine)	
		Preexisting headache: Migraine = 26.2%, TTH = 26.7%							
4	Ozge Uygun et al. Turkey, Istanbul. 2020. A Survey study based on a web-based questionnaire in two groups: Group 1) COVID positive headache group and, Group 2) non-diagnosed COVID patients with new headaches in the pandemic. It’s a cross-sectional design. N = 3458	Headache, n = 1886	Bilateral: 85% in the COVID group and 64% in the non-COVID group	In COVID group new onset headache: Pulsating = 32.5%, pressing = 43.7%, stabbing = 16.1%, Fiery = 2.3%	In COVID and non-COVID group: a/w anosmia/ageusia = 60.4%, gastrointestinal upset = 57.7% (diarrhea, stomachache, nausea), phonophobia, photophobia, allodynia, osmophobia (reported only in COVID group)	Overall course of headache: Among 1886 patients Increase in severity = 23.3% Decreased in severity = 72.4% Increase in duration = 28.7% Decreased in duration = 66.4% Increase in frequency = 28.7% Decrease in frequency = 12.3% Associated symptoms increased = 14%	No	Chance to study headache of COVID and non-COVID patients here. Amongst previous headache history, 50% experienced a totally different quality in a new headache	[6]
		Mean age = 43.21±11.2		In non-COVID headache: Pulsating = 42.5%, pressing = 38.6%, stabbing = 11.2%, Fiery = 5%	Triggers: (In decreasing % trend) in COVID group: stress, Infection itself, drugs, wearing masks, social isolation	If a prior history of headache is present in both groups, now, a pulsating quality headache is seen in 50.9% and 55.5%, respectively		A drawback of the study is the reporting bias, the patient was not examined; educated people could participate because of the web-based survey	
		Male in the COVID group = 40%, Male in non-COVID group = 25%		Intensity in COVID headache: Mild = 26.6%, Moderate = 47.7%, Severe = 23.4%	Triggers in the non-COVID group: Stress, social isolation, and wearing masks			Higher risk for male gender; Causes could be: 1) protective female hormones have anti-inflammatory action 2) ACE-2 location could be speculated in X chromosomes efficient immune activity in females and ACE-2 expression different	
				Intensity is almost similar to a non-COVID headache.				Gastrointestinal symptoms: Related to gut-brain axis related to inflammatory mediators like IL-1, IL-6, IL-8, TNF-alpha, gut microbiota, gut CGRP	
								22.5% of COVID-19-positive patients, who had a previous history of headaches, did not experience any headaches when infected. No clear reason was found but could be attributed to low viral load or individual differences	
5	Tugce Toptan et al. Turkey. 2020. Case series of 13 patients. N = 13	Headache patients, n = 13	Holocranial, bilateral frontal, and temporal areas	Acute onset, Throbbing quality, superimposed with stabbing and pressing character	Photophobia, phonophobia, nausea, osmophobia, weight loss	No	No scans were done.	Previous migraineurs described the associated symptoms were profound compared to the previous experience of headaches prior to viral infection	[7]
		Female = 9/13		The intensity of the headache was moderate to severe	Aggravated by movements and bending forward			All headaches were different from the previous headache history. Partial response to NSAIDs or paracetamol was seen. 9/13 patient’s headache recovered in 72 hours	
		Out of that 5/13 had a history of migraine. 1/13 had a history of TTH. 2/13 (case 4 and case 7) had a history of DM						Significant weight loss (5 kg in 3 days) was seen, not related to diarrhea or loss of appetite, which was for 1 day, could be related to catabolism attributed to cytokines or cortisol levels	
6	Pedro Augusto et al. Brazil. 2020. Case report. N = 1	Female	The headache started on the 5^th^-day of illness. b/l frontotemporal	Pulsating character. Duration= 7 days, continuous and severe intensity	4^th^ day had anosmia with facial pain that lasted for 48 hours	Pt says intra-covid headache was different and most disturbing from her pre-COVID headaches, although the character was similar to migraine	No scans done	Headache not related to intake of painkillers and despite their use, headache improved gradually so medication overuse headache (MOH) is ruled out here but can be classified as probable new daily persistent headache (NDPH) since this COVID headache lasted for about 3 months	[8]
		40 years			5^th^-day headache a/w photophobia and phonophobia and increased with mild movements			On follow-up post 85 days of COVID infection and in current follow-up, persistent headache and anosmia are present. Headache lasts for 6 hrs, moderate to severe intensity, frequency not mentioned	
		Past history of migraine with and without aura						No lumber puncture was done. MRI, MRA, and MRV were normal, with no contrast enhancement (this scan was done 23 days after the appearance of the first symptom)	
7	Robert Belvis. Barcelona, Spain. 2020. Case report. N = 1	Male. Neurologist by profession, a headache expert	Posterior to diffuse	Headache phenotypes in first 3 days: 1^st^ Day: Episodic met ICHD3 criteria (code9.2.2.1) diffuse pain, moderate intensity, related to fever, responded well to acetaminophen	Anosmia, dysgeusia for the first 3 days	On the 7^th^ day: the reappearance of a new headache. Diffuse location, continuous with moderate intensity with mild neck stiffness and photophobia	Acute phase reactant: D-dimer, ferritin, procalcitonin, and CRP were high but lymphopenia with low platelet count	Possible triggers for TTH could be a cervicogenic factor due to the use of mobile phones in bed, stress due to anxiety of bad future prognosis of disease, insomnia due to anxiety	[9]
		Age = 51 years		2^nd^ Day: primary cough headache (code 9.2.2.1), sudden onset, lasted 2 minutes, posterior and b/l location, responded well to above medicine	On day 7: associated with neck stiffness and photophobia	7^th^-day headache: Worsened with posture change and physical activity attributed to hypoxia and hypercapnia (code 10.1)		7^th^-day headache could be a consequence of a cytokine storm more of an aseptic meningeal activity and could be included in ICHD3-Headache attributed to other non-infectious inflammatory intracranial disease (code 7.3.3) or attributed to exposure to other substances (code 8.1.11) like cytokines	
				3^rd^ Day: pressing b/l headache (code2) of Tension-type headache, mild intensity, progressive, tenderness in the sternocleidomastoid, splenius, and trapezius					
8	Javier Trigo et al. Spain. 2020. Cohort design. Retrospective study. N = 580.	Headache, n = 137/580, No headache, n = 170/580	No detailed descriptions were given for the location and quality of the headache	Headache as the 1^st^ symptom seen in 26% of patients	Headache associated with anosmia = 46.7% and syncope = 6.6%	no	Statistically significant mean values in terms of laboratory parameters in both groups, where the ‘No headache’ group had larger values mentioned below: Worst leucocyte, lymphocyte, LDH count, CRP, Procalcitonin, ferritin, IL-6, D-Dimer, worst INR values	Interpreted headache as a common symptom of COVID-19 and carries lower mortality risk, an independent variable. Similar results of no worse prognosis with COVID-19 headache patients in a meta-analysis of 19 studies done at the beginning of 2020	[4]
		Mean Age = 59.25 years; Female = 58.4%		Headache seen: Within 1 day = 38.5%, Within 2 days = 62.5%, Within 3 days = 74%	Non headache group: associated with anosmia = 18.7% and syncope = 7.7%		No brain scans done	Lower inflammatory markers probably could not initiate a storm of cytokines, resulting in less mortality	
		Co-morbids: HTN = 38%, DM = 14.6%, Smoking = 14.6%, CVS = 13.9%, Pulmonary = 24.1%, Cancer = 13.1%, Prior headache = 10.9%, ACEi or ARBs = 30.7%						Drawbacks include a lack of imaging and CSF studies. Single center study. The sample included only hospitalized patients. A retrospective study where relatives were also contacted instead of patients. Detail about headache character not mentioned	
								In another prospective cohort study with 179 hospitalized COVID-19 patients, mortality % recorded with headache was more (23.8%) compared to those without a headache	
9	David et al. Spain. 2020. Cross-sectional study, hospitalized patients. N = 576	Headache 22.6% (130/576) but 104 included in the study	Location and quality mentioned in the red flags of headache part	1^st^ COVID symptom as headache noted in 26.0% of patients	Anosmia= 64.4%, AMS= 9.6%, Weakness= 1.9%, Vertigo= 2.9%, Loss of consciousness= 5.8%	Red flags of headache were present in 95.2% of patients and the most frequent was a change in the pattern of preexisting headache, seen in 49.0%	Blood investigations: Lymphopenia = 21.1%, increased LDH = 48%, INR = 8.7%, D-Dimer = 54%, CRP = 84%, Procalcitonin = 3.8%	The most common atypical presentation of headache in the form of red flags is pattern change in headache = 49%, recent onset of headache = 42.3%, Worst headache = 37.5%. (Could be false high and related to prior headache history)	[10]
		Mean age = 67.6 yrs± 12.2		Red flags of headache were inquired about Prior medical history: Onset>50 years = 72.1%, h/o cancer = 12.5%, Immune-compromised state = 4.8%.			No scans done	Others being related to systemic factors like Fever= 89.4%, cough= 84.6%, raised CRP= 84%	
		Female = 74.1%		Red flags of headache were inquired about Recent symptoms related to headache: precepting factors = 37.5%, thunderclap onset = 4.8%, change in pattern = 49%, progressive worsening = 17.3%, ocular pain = 30.8%, any cranial autonomic features = 5.8%, positional pattern = 6.7%, interrupting sleep = 16.3%, worst headache ever = 37.5%, unilaterality = 15.4%, treatment resistance = 14.4%				Limitations included hospitalized patients with severe symptoms, here in this study 95% had pneumonia	
		Co-morbid states: HTN = 34.6%, DM = 11.5%, Smoking = 11.5%, CVS = 8.7%, Pulmonary= 23.1%, Cancer= 12.5%, Prior headache= 57.7%, Migraine= 16.3%, TTH= 28.8%, Family h/o headache= 37.5%, On ACEi or AT-II= 28.8%						CSF studies and scans were not conducted due to a shortage of equipment	
								Not focused on phenotype	
10	Omer Karadas et al. Turkey. October 2020. Cross-sectional prospective study. N=287	Headache, n= 28.9% (83/287). Out of 83, 85.5% had no complaint of headache	Frontal= 54%, Occipital= 15.6%, Frontotemporal= 3.6%, Unilateral= 7.2%, Bilateral= 92.8%	Throbbing= 56.6%, Pressing= 25.3%, Stabbing= 8.4%.	Hyposmia= 8.4%, agneusia= 7.2%, Photophobia= 30.1%, Phonophobia= 27.7%. Others were loss of appetite, weight loss, and diarrhea	no	D-dimer was high in the headache group	b/l headache was prominent in frontal regions and had higher IL-6 and D-dimer levels. Throbbing quality had a better response to medicines. Loss of appetite, weight loss, and diarrhea was high in the headache group (could be related to CGRP neuro-peptide released in both sites, gastrointestinal and nervous system)	[11]
		Gender: male (58% of headache patients were male)						Intravenous paracetamol 1000mg, greater occipital nerve block (GON) using 2% lidocaine were used. High IL-6 levels and pneumonic patients had higher VAS scores. Patients unresponsive to medical treatment were young, had a median age of 40 years, and had high levels of IL-6	
		Co-morbid states in headache group: HTN= 39.8%, DM= 14%, CAD= 9.6%						Patients responsive to GON block were young and again unresponsive patients had a high IL-6 level	
								In this study, low hyposmia rates of 5% and a good recovery rate were noted, suggesting hyposmia is not an outcome predictor	
11	Javier et al. Madrid, Spain. 2020. Cross-sectional study in the emergency department. N =145. Probable COVID-19 patients since the study was done in the emergency department	Headache 68.3% (99/145)	Bilateral = 86.9%, Holocranial = 34.3%, Bifrontal = 34.3%	Pressing = 73.7%, Throbbing = 14.1%, Stabbing = 11.1%, Burning = 1%	Anosmia = 49.5%, light aversion = 29.3%, phonophobia = 27.3%, osmophobia= 9.1%	no	No	Noted diverse headache semiology. Migraineurs had more-intense presents earlier and had a longer headache	[12]
		Mean Age 42.7±11.5 (Range: 21 to 70 yrs)		Moderate to severe intensity	Triggers were afternoon fever, physical activity, and coughing			No CSF studies were done. Could not rule out SARS-CoV-2 meningitis. The patient experienced different headache quality when compared to previous ones	
		Female = 36.4%		One patient had trigeminal autonomic findings of eyelid edema and otic fullness with ipsilateral headache and One patient had visual aura					
		headache history = 33.3%, Hypertension = 15.2%, DM = 3%, Dyslipidemia = 11.1%							

Olfactory and gustatory afflictions perceived with COVID-19 are profound at about 52.7% and 43.9%, respectively [5]. Anosmia/hyposmia, ageusia/hypogeusia, and phonophobia are frequently related to COVID-19 headaches [3,5]. However, these sensory findings propose a satisfactory prognostic marker as evidenced by almost a week shorter COVID-19 disease span/hospital stay [3]. Collaboratory magnetic resonance imaging (MRI) brain scans may divulge hyperintensity in the olfactory bulb [5] and gyrus rectus (a segment of the medial inferior frontal lobe) [7], which was unfortunately not conducted in our cases.

Hypothalamus: a possible ringleader

During a cluster/trigeminal autonomic cephalalgia (TAC) headache attack, the posterior hypothalamus gets activated [13], whereas an early morning attack might disturb the hypothalamic-pituitary axis. This axis is presumably related to an altered melatonin and cortisol level [14]. It is noteworthy that the supraoptic nucleus, paraventricular nuclei, and infundibular nuclei of the hypothalamus suggest a possible role in cluster headache (CH) pathophysiology. These nuclei have the highest content of calcitonin G-related peptide (CGRP) neurons, particularly in the RAMP1 loci as evidenced by previous experiments conducted on mice [14]. The hypothalamus regulates the hormonal cycle essential for maintaining internal homeostasis correlating with cyclic and circadian cluster phenotype, where the usage of deep brain stimulation (DBS) is a feasible treatment option for intractable cluster headaches [13].

Even in migraine, the genesis locus shifts from brainstem regions viz. periaqueductal gray (PAG), dorsal raphe (DR), and locus coeruleus (LC) to anterior and posterior hypothalamus, serving as the initiation of pain attack [13]. Surprisingly, these regions are found to be active 24 hours prior to migraine pain [15]. Coupling with dorsal pons and trigeminal nucleus caudalis (TNC), the hypothalamus has become a supreme generator for an attack while expressing circadian clock genes. Hypothalamic perturbance can be a possible phenomenon for abnormal headache experiences and requires further validation.

The proposed possible pathomechanism

**Figure 1:**
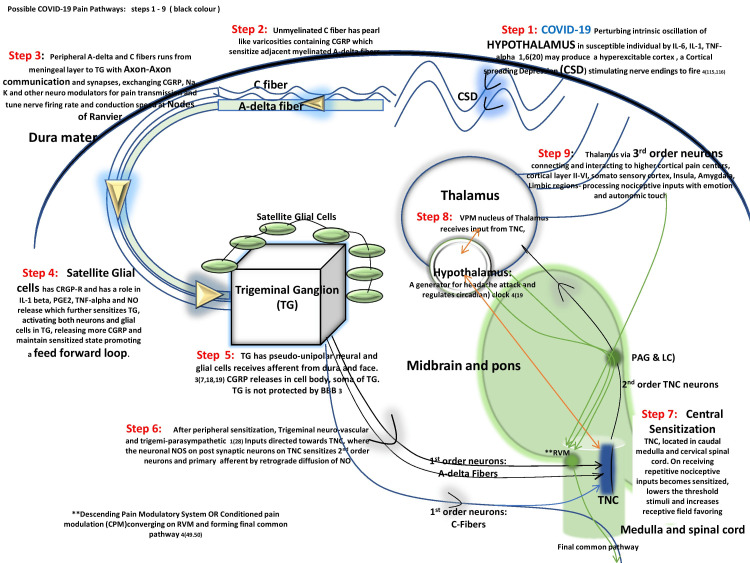
Possible COVID-19 pain pathway engaging IL-1, IL-6, and TNF-alpha to initiate the cascade. CSD stimulates cortical nerve endings perturbing the hypothalamus in susceptible individuals generating headache TG: trigeminal ganglion; CSD: cortical spreading depression; IL: interleukin; TNF: tumor necrosis factor; PGE2: prostaglandins E2; NO: nitric oxide; CGRP: calcitonin G-related peptide; TNC: trigeminal nucleus caudalis; VPM: ventral posteromedial Source: Original illustration

Fever escalates pro-inflammatory cytokines, viz. interleukin-1 (IL-1) and interleukin-6 (IL-6), agitating the hypothalamus and further ascribing to headache [1]. Contrarily, fever-free headaches have also been reported [1,3], thereby suggesting the possibility of some other pathways involved with such headaches. COVID-19 triggers a cytokine storm of IL-6, which is well known for causing neuroinflammation in migraineurs [3]. Connecting these two events raises the possibility of migraine headache mechanistic with COVID-19 sufferers (illustrated in Figure 1); however, surprisingly stable or even lower levels of IL-6 are observed with COVID-19 headaches [3]. These stable levels might act as a protective gesture attempting to keep the neuroinflammation at a localized level [3]. TNF-alpha has a role in the peripheral and central sensitization of pain and is detected in higher concentrations at the neuropathic pain site [16]. It is unknown whether TNF-alpha has any role in COVID-19 headache physiology, and this is a major topic of research.

It is well known that cortical spreading depression (CSD), a slow-moving depolarization wave (3-5 mm/ min) secondary to cortical hyperexcitability, initiates centrally and has an impact on the descending pain modulatory system leading to peripheral and delayed central sensitization as illustrated in Figure 1. It is a debatable theory whether CSD stimulates meningeal C fibers to release CGRP and reactive oxygen species (ROS) or whether its action on TNC neurons produces headache and aura [13]. The above theory could be a possibility explaining the headache in COVID-19 patients without aura, as seen with migraineurs without aura.

**Figure 2:**
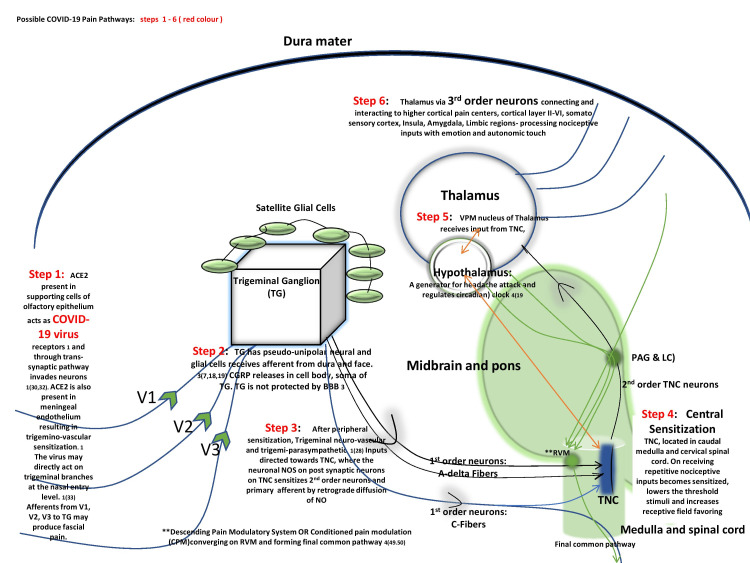
Possible COVID-19 pain pathway. Neurotropic COVID-19 virus attempts to capture peripheral nerve terminals (CN5). In the process, it adheres to ACE2 activating the trigeminovascular system to interact with higher cortical pain centers via thalamic and hypothalamic nuclei TG: trigeminal ganglion; NO: nitric oxide; TNC: trigeminal nucleus caudalis; CN5: cranial nerve 5; VPM: ventral posteromedial; ACE2: angiotensin-converting enzyme 2; IL: interleukin; TNF: tumor necrosis factor; PGE2: prostaglandins E2; NO: nitric oxide; CGRP: calcitonin G-related peptide. Source: Original illustration

The COVID-19 virus may have a neurotropic disposition, capturing peripheral nerve terminals (cranial nerve 5) for using the trans-synaptic pathways to acquire entry to the CNS [3,6]. This occurs via the nasal cavity through the olfactory bulb to the piriform cortex, employing passive diffusion in axonal transport [6] (displayed in Figure 2). Viruses adhere to angiotensin-converting enzymes2 (ACE2), expressed on the specialized olfactory epithelium, thereby invading the olfactory nerve. This adherence could also be via direct annexation of the peripheral branches of the trigeminal at the nasal epithelium. Even a modus operandi of using both paths can finally activate the trigeminovascular system peripherally [3]. It is noteworthy that ACE2, a transmembrane metalloproteinase, is tremendously expressed in supporting cells of the olfactory epithelium, as well as in the meningeal endothelium, where it downregulates itself on binding to SARs-CoV-2 [17]. This disturbs the AngI-ACE-AngII-AT1R axis [7], ultimately raising levels of angiotensin1 receptor (AT1R) and calcitonin G-related peptide (CGRP), thereby contributing to headaches [17].

**Figure 3:**
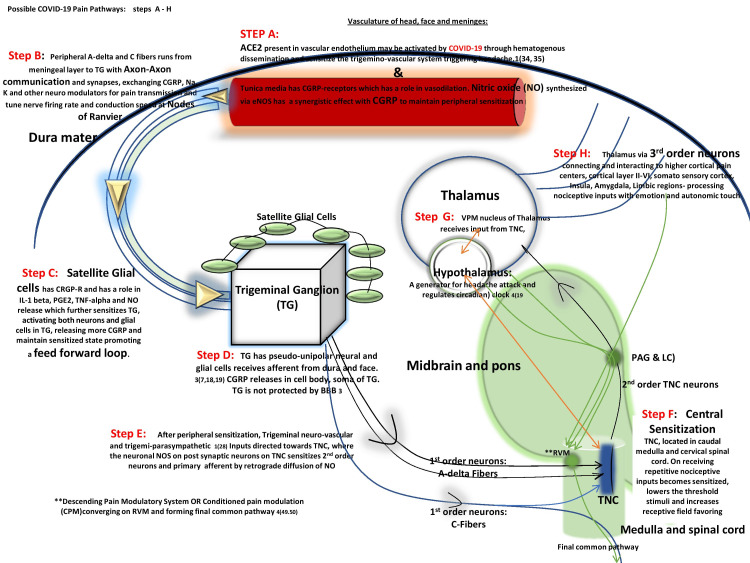
Possible COVID-19 pain pathway. SARs-CoV-2 binds to ACE2 present in intracranial vascular endothelium sensitizing the trigeminovascular system TG: trigeminal ganglion; NO: nitric oxide; TNC: trigeminal nucleus caudalis; CN5: cranial nerve 5; VPM: ventral posteromedial; ACE2: angiotensin-converting enzyme 2 Source: Original illustration

ACE2 acts on angiotensin II (Ang II) generating angiotensin 1 to 7 (Ang1-7), which serves good functions in the body such as neuroprotection, decreasing blood pressure, lowering reactive oxygen species, and preventing neuro-degeneration, etc. [17]. The disservice of AT1R and benevolence of Ang1-7 are present throughout the body. AT1R is present in peri aqueductal gray matter (PAG), anterior cingulate cortex (ACC), prefrontal cortex, and the spinal cord. Not only do they modulate nociceptive pain but also impart an emotional dimension through the amygdala [16]. As soon as SARs-CoV-2 binds to ACE2, AT1R is favored in the body, generating CGRP and headache. ACE2 can sensitize the trigeminovascular system (Figure 3), which may persist even if the culprit pathogen is treated, thereby leading to headache chronification [3].

The final common pathway from the rostral ventromedial medulla RVM, forming descending pain modulatory system has a robust conditioned pain modulation (CPM) in healthy subjects, which causes descending pain inhibition when activated [13]. A recent study on episodic and chronic migraineurs showed reduced and no CPM, respectively [13], enforcing the concept of loss of descending pain inhibition and could be rather facilitating for the trigeminovascular system [13]. This could be the reason behind the increased frequency of headache attacks seen in COVID patients who had a prior history of primary headache disorder as noted in another study from Egypt [1]. Fever, myalgia, and profound cytokines release due to COVID-19 could alter CPM and may behave as a facilitator of descending pain inhibition. Localized periorbital cutaneous allodynia driven by central sensitization at TNC-level neurons and widespread allodynia suggest a third-order thalamic level [13]. Facial and head cutaneous sensory symptoms of burning and discomfort due to COVID-19 may be hypothesized as a result of central sensitization of repetitive insult by virus elements or headache itself.

The overlapping concept of immunotherapy-induced chimeric antigen receptor (CAR) T cell neurotoxicity and cerebral edema presenting as intractable headaches might bear some similarities with COVID-19 headaches [2]. Even intracranial cytokine storm-breaking BBB, as hypothesized in one recent case report of acute viral necrotizing encephalopathy, could be a possible pathomechanism [2].

## Conclusions

This pandemic was able to act as a guiding tool for mankind for a comprehensive understanding of the enigmatic concepts regarding headaches. Our case report is useful not only in the current chaotic pandemic but also in potential future waves, as this virus can be easily transmitted by aerosol Flugge microdroplets. Drugs such as tocilizumab, known to treat giant cell arteritis, also play an important role in modulating cytokine in COVID-19. Moreover, intranasal Vazegepant, a CGRP receptor antagonist for acute migraine, has a promising ongoing trial for COVID-19 lung infection treatment and suggests a possibly complex connecting link of the headache pathophysiology to the above ailments. Multicentric and multinational studies should be conducted for outlining the COVID-19 headache phenotype and trend since enough disparity from previous reports can be seen, as outlined in Table 1. Treatment coverage plans and policies by the health insurance companies in this COVID-19 situation must also be considered to ensure that the cost and health burden for our headache patients can be minimized. This would ease their attendance in clinics, where clinicians can try various headache procedures and utilize newer injectables or devices. This is the right time to strike to acquire a better understanding with scrutiny for this centuries-old ailment.
